# Neonatal mortality risk of large‐for‐gestational‐age and macrosomic live births in 15 countries, including 115.6 million nationwide linked records, 2000–2020

**DOI:** 10.1111/1471-0528.17706

**Published:** 2023-11-27

**Authors:** Lorena Suárez‐Idueta, Eric O. Ohuma, Chia‐Jung Chang, Elizabeth A. Hazel, Judith Yargawa, Yemisrach B. Okwaraji, Ellen Bradley, Adrienne Gordon, Jessica Sexton, Harriet L. S. Lawford, Enny S. Paixao, Ila R. Falcão, Sarka Lisonkova, Qi Wen, Petr Velebil, Jitka Jírová, Erzsebet Horváth‐Puhó, Henrik T. Sørensen, Luule Sakkeus, Lili Abuladze, Khalid A. Yunis, Ayah Al Bizri, Sonia Lopez Alvarez, Lisa Broeders, Aimée E. van Dijk, Fawziya Alyafei, Mai AlQubaisi, Neda Razaz, Jonas Söderling, Lucy K. Smith, Ruth J. Matthews, Estelle Lowry, Neil Rowland, Rachael Wood, Kirsten Monteath, Isabel Pereyra, Gabriella Pravia, Joy E. Lawn, Hannah Blencowe, the National Vulnerable Newborn Mortality Collaborative Group and the Vulnerable Newborn Measurement Core Group, Vicki Flenady, Kara Warrilow, Harriet Lawford, Mauricio Lima Barreto, Ila Rocha Falcão, Erzsébet Horváth‐Puhó, Liili Abuladze, Pascale Nakad, Arturo Barranco Flores, Jesus Felipe Gonzalez Roldan, Fawzia Alyafei, Tawa O. Olukade, Hamdy A. Ali, Mohamad Rami Alturk, Bradley N. Manktelow, Elizabeth Draper, Alan Fenton, Jennifer J. Kurinczuk, Celina Davis, Samantha Clarke

**Affiliations:** ^1^ Mexican Society of Public Health Mexico City Mexico; ^2^ Maternal, Adolescent, Reproductive & Child Health (MARCH) Centre, London School of Hygiene & Tropical Medicine London UK; ^3^ Department of International Health Johns Hopkins Bloomberg School of Public Health Baltimore Maryland USA; ^4^ Faculty of Medicine and Health University of Sydney Camperdown New South Wales Australia; ^5^ National Health and Medical Research Council (NHMRC) Centre of Research Excellence in Stillbirth, Mater Research Institute, The University of Queensland Brisbane Queensland Australia; ^6^ Center for Data and Knowledge Integration for Health (CIDACS) Instituto Gonçalo Moniz, Fiocruz Bahia, Fundação Oswaldo Cruz Salvador Brazil; ^7^ Department of Obstetrics & Gynaecology University of British Columbia Vancouver British Columbia Canada; ^8^ Department of Obstetrics and Gynaecology Institute for the Care of Mother and Child Prague Czech Republic; ^9^ Department of Data Analysis Institute of Health Information and Statistics of the Czech Republic Prague Czech Republic; ^10^ Department of Clinical Epidemiology Aarhus University and Aarhus University Hospital Aarhus Denmark; ^11^ School of Governance, Law and Society, Estonian Institute for Population Studies Tallinn University Tallinn Estonia; ^12^ The National Collaborative Perinatal Neonatal Network (NCPNN) Coordinating Center at the Department of Pediatrics and Adolescent Medicine American University of Beirut Beirut Lebanon; ^13^ Perined Utrecht the Netherlands; ^14^ Hamad Medical Corporation Doha Qatar; ^15^ Clinical Epidemiology Division, Department of Medicine Solna Karolinska Institutet Stockholm Sweden; ^16^ Department of Population Health Sciences, College of Life Sciences University of Leicester Leicester UK; ^17^ School of Natural and Built Environment Queen's University Belfast Belfast UK; ^18^ Queen's Management School Queen's University Belfast Belfast UK; ^19^ Usher Institute Edinburgh UK; ^20^ Pregnancy, Birth and Child Health Team, Public Health Scotland Edinburgh UK; ^21^ Faculty of Health Sciences Catholic University of Maule Curicó Chile; ^22^ Department of Wellness and Health Catholic University of Uruguay Montevideo Uruguay

**Keywords:** fetal macrosomia, infant, large for gestational age, neonatal mortality, pregnancy

## Abstract

**Objective:**

We aimed to compare the prevalence and neonatal mortality associated with large for gestational age (LGA) and macrosomia among 115.6 million live births in 15 countries, between 2000 and 2020.

**Design:**

Population‐based, multi‐country study.

**Setting:**

National healthcare systems.

**Population:**

Liveborn infants.

**Methods:**

We used individual‐level data identified for the Vulnerable Newborn Measurement Collaboration. We calculated the prevalence and relative risk (RR) of neonatal mortality among live births born at term + LGA (>90th centile, and also >95th and >97th centiles when the data were available) versus term + appropriate for gestational age (AGA, 10th–90th centiles) and macrosomic (≥4000, ≥4500 and ≥5000 g, regardless of gestational age) versus 2500–3999 g. INTERGROWTH 21st served as the reference population.

**Main outcome measures:**

Prevalence and neonatal mortality risks.

**Results:**

Large for gestational age was common (median prevalence 18.2%; interquartile range, IQR, 13.5%–22.0%), and overall was associated with a lower neonatal mortality risk compared with AGA (RR 0.83, 95% CI 0.77–0.89). Around one in ten babies were ≥4000 g (median prevalence 9.6% (IQR 6.4%–13.3%), with 1.2% (IQR 0.7%–2.0%) ≥4500 g and with 0.2% (IQR 0.1%–0.2%) ≥5000 g). Overall, macrosomia of ≥4000 g was not associated with increased neonatal mortality risk (RR 0.80, 95% CI 0.69–0.94); however, a higher risk was observed for birthweights of ≥4500 g (RR 1.52, 95% CI 1.10–2.11) and ≥5000 g (RR 4.54, 95% CI 2.58–7.99), compared with birthweights of 2500–3999 g, with the highest risk observed in the first 7 days of life.

**Conclusions:**

In this population, birthweight of ≥4500 g was the most useful marker for early mortality risk in big babies and could be used to guide clinical management decisions.

## 1 INTRODUCTION

Globally, substantial focus has been placed on babies who are small at birth, because of their elevated risks of neonatal morbidity and mortality, and the associated long‐term health implications.^1^, ^2^, ^3^, ^4^, ^5^ However, the effects of being large for gestational age (LGA) at birth have received relatively less attention, despite this condition has been also associated with maternal and perinatal morbidity.^6^, ^7^, ^8^, ^9^, ^10^ Maternal complications include prolonged labour, increased rate of caesarean section, perinatal trauma, postpartum haemorrhage and uterine rupture,^11^ whereas large babies are at higher risk of shoulder dystocia, brachial plexus injury, fractures, hypoglycaemia and prolonged hospitalisation.^8^ Later in life, being born LGA has been associated with an increased risk of being overweight, obese, or suffering from psychiatric conditions, diabetes, hypertension or cancer, in childhood and adulthood.^11^, ^12^, ^13^

Large size at birth may result from excessive fetal weight gain during pregnancy or from prolonged pregnancy.^6^, ^14^, ^15^ Commonly used measures for large infants include the following: LGA, defined as birthweight above the 90th centile for sex and gestational age; and ‘macrosomia’, defined as birthweight above 4000 g, regardless of gestational age.^16^ LGA include subgroups of constitutionally large and overnourished babies who experience different risks of clinical complications.^17^, ^18^

To date, no worldwide systematic assessment of the proportion of babies with large size at birth has been performed. Existing assessments of the proportion of LGA babies at the population level may differ according to maternal, social and environmental factors, as well as the growth chart used.^6^, ^19^ By definition, approximately 10% of babies are found to be LGA when national descriptive growth charts are used.^20^, ^21^, ^22^ However, when prescriptive international standards are used, the prevalence of LGA can vary widely among populations. Using the INTERGROWTH‐21st standards, previous studies have reported LGA rates of 8.0%–25.1% in Australia and in 16 European cohorts.^20^, ^21^, ^22^, ^23^, ^24^ Published prevalences of macrosomia have ranged from 5% to 20% in high‐income countries and from 1% to 14.9% in low‐ and middle‐income countries.^25^, ^26^

Although perinatal outcomes associated with large babies have been studied, whether these medical complications contribute to early mortality, and which cut‐offs are most predictive of neonatal death, remain unclear. Studies have suggested that subgroups of macrosomia (≥4000, ≥4500 and ≥5000 g) could better identify newborn vulnerability and risk.^6^, ^14^, ^27^

**FIGURE 1:**
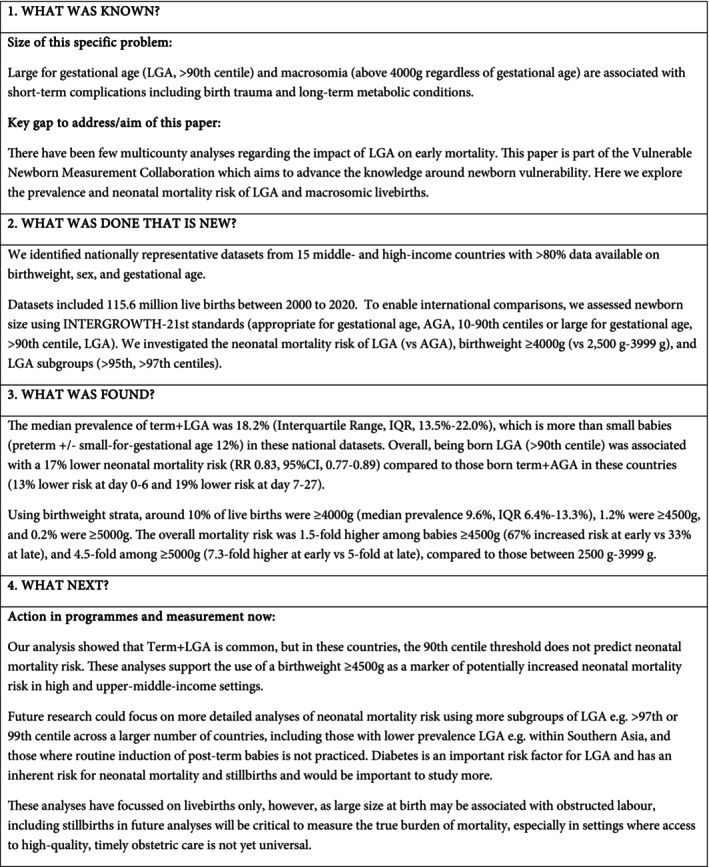
Key findings.

This article is one of a series aimed at advancing the assessment and measurement of newborn vulnerability that propose a set of six newborn types, combining gestational age (term, T, versus preterm, PT) and newborn size (small for gestational age, SGA; appropriate for gestational age, AGA; or LGA), and using the INTERGROWTH 21st international standards as the reference population.^28^, ^29^, ^30^ Herein, we aimed to quantify the prevalence and neonatal mortality risk of large babies by exploring the groupings by newborn type (term + LGA vs term + AGA) in objective 1 and by birthweight (≥4000, ≥4500 and ≥5000 g, regardless of gestational age, vs a comparison group weighing 2500–3999 g) in objective 2 (Figure 1).

## 2 METHODS

### 2.1 Compilation of data sets

Collaborators and governmental agencies with national individual‐level data sets with high population‐level coverage (including more than 80% of births in each country) were invited to participate in a collaboration, focusing on the multi‐country description of types of vulnerable newborn babies. An open call was published in a *Lancet* commentary,^31^ and was widely disseminated through email lists, social media and by contacting authors of previously published analyses using national routine administrative data sets. A detailed description of how the data were extracted and handled can be found elsewhere.^32^

Among the national data sets identified in the Vulnerable Newborn Measurement Collaboration, this analysis considered 15 national data sets with electronic records of births and neonatal deaths collected between 1 January 2000 and 31 December 2020.^33^ Relevant definitions and variables are summarised in Table S1. This was an analysis of routinely collected data; therefore, we followed the Reporting of Studies Conducted Using Observational Routinely Collected Data (RECORD) guidelines (Table S2). Ethical approvals for all 15 participating countries are compiled in Table S3.

### 2.2 Data quality, and inclusion and exclusion criteria

The data quality of 15 national data sets was examined by quantifying the number of missing values for three core variables (birthweight, gestational age and sex) used to assess newborn size (Table S4). Reporting practices and metadata are summarised in Table S5. We included national data sets with information on live births and deaths with high completeness (at least 80%) for birthweight, gestational age and sex of the child.

Birth records with implausible values for birthweight (<250 g or ≥6500 g) or gestational age (<22 weeks or >44 weeks), or with implausible combinations of birthweight and gestational age (defined as birthweight ±5 standard deviations from the mean birthweight at each completed week of gestational age) were excluded.

### 2.3 Exposure and outcome definitions

To fulfil objective 1, we assessed the size of the newborn using an extended version of the INTERGROWTH‐21st standards from 22^+0^ to 44^+6^ weeks of gestation (Figure S1a).^22^, ^28^, ^29^ Each baby was categorised by combining the size at birth (defined as SGA, <10th centile; AGA, between 10th and 90th centiles; or LGA, >90th centile) and gestational age (defined as preterm, <37 weeks of gestation; or term, ≥37 weeks of gestation including post‐term births up to 42 weeks of gestation). Given that this analysis only focuses on those at term + LGA and term + AGA, we excluded babies born preterm or SGA (Figure S2).

To fulfil objective 2, we categorised each live birth according to the recorded weight at birth as macrosomic (using three cut‐offs: ≥4000, ≥4500 and ≥5000 g) or low birthweight (<2500 g). The comparison group included babies weighing 2500–3999 g. Live births with weights of <2500 g were excluded.

To estimate mortality risks, neonatal survival status was reported at 28 days after delivery. We defined neonatal deaths as deaths occurring from 0 to 27 days after a live birth. Neonatal deaths were further classified into early (0–6 days) or late (7–27 days).^16^

### 2.4 Statistical analysis

Each country's team analysed national data sets by using standard code in STATA (StataCorp, College Station, TX, USA), SAS (SAS Institute, Cary, NC, USA) or R programming languages developed centrally by the London School of Hygiene & Tropical Medicine (LSHTM). Standard summary tables were shared in a hub administered online by LSHTM.

We calculated prevalence by dividing the number of live births in the group of interest by the total number of live births reported per 100. Neonatal mortality rates (NMRs) were defined as the number of live births experiencing the event (neonatal death) divided by the total number of live births exposed to the risk of that event per 1000. Crude relative risk (RR) values and corresponding 95% confidence intervals (95% CIs) were calculated as the risk in each exposure group of interest divided by the risk in the reference group (e.g. LGA vs AGA and macrosomic vs 2500–3999 g). To describe which thresholds of macrosomia are most predictive for neonatal mortality risk, we calculated relative risk using three levels of exposure: ≥4000, ≥4500 and ≥5000 g.

We performed a secondary analysis including livebirth subgroups AGA (10–90th centiles) and LGA (>90th, >95th and >97th centiles) occurring at each gestational age in four countries where more detailed information regarding newborn size by centiles was available (Figure S1b). We quantified the neonatal mortality risk of LGA babies compared with their AGA counterparts born during the same gestational week.

The overall prevalence and NMR were summarised using medians and interquartile ranges. Additionally, we explored early (0–6 days), late (7–27 days) and neonatal (0–27 days) mortality risks, pooling results using a random effects meta‐analysis in view of the substantial heterogeneity observed across countries.^34^, ^35^

## 3 RESULTS

### 3.1 Prevalence and neonatal mortality risk associated with LGA

**FIGURE 2:**
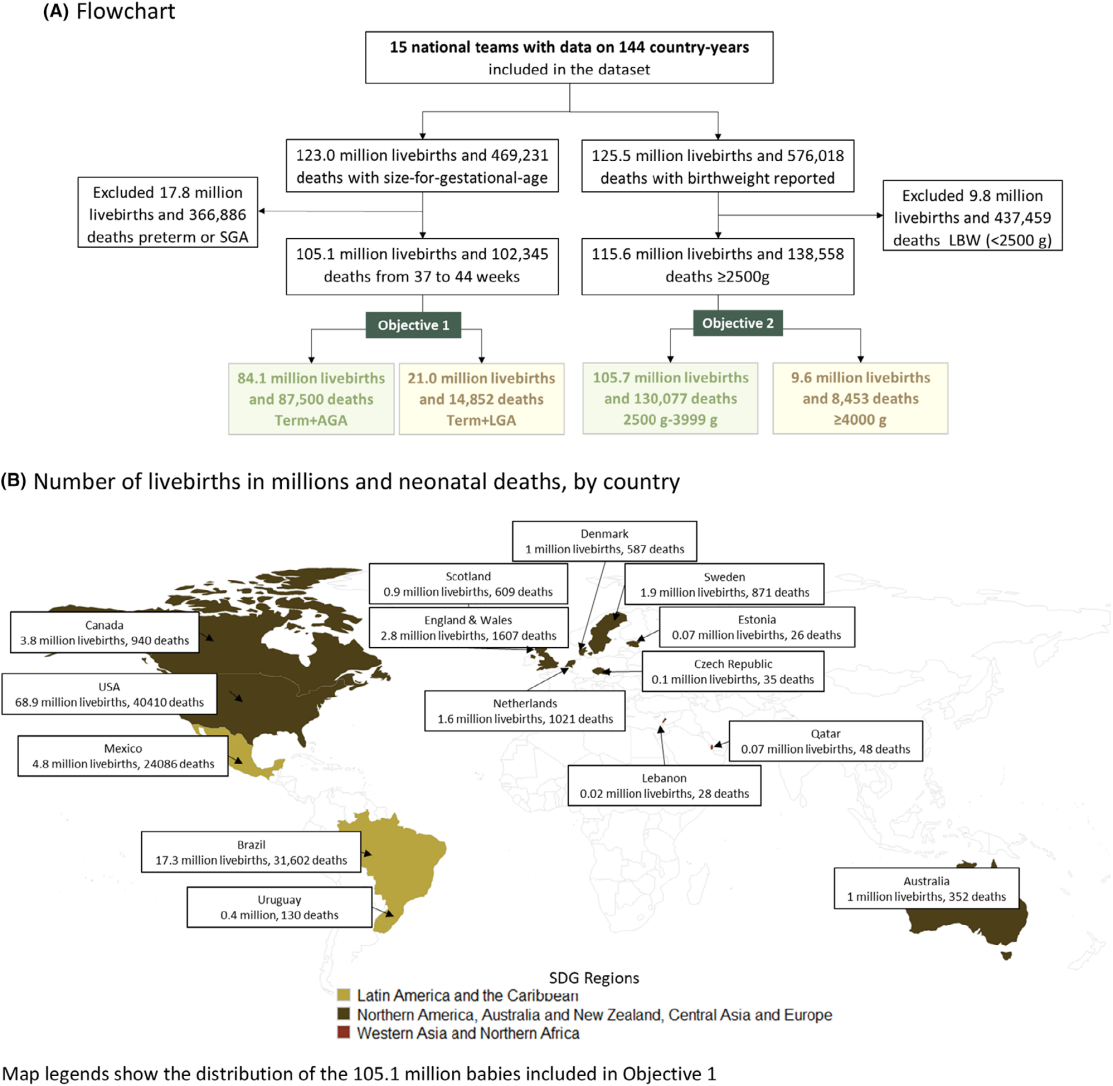
Input data set of the Vulnerable Newborn Mortality study. (A) Flowchart. (B) Number of live births in millions and number of neonatal deaths, by country.

Data were collated from 15 countries, representing three Sustainable Development Goals regions (Latin America & the Caribbean; North America, Australia, New Zealand, Central Asia & Europe; and Western & North Asia), totalling 144 country‐years from 2000 to 2020. We identified 123 million live births and 469 231 neonatal deaths with information on size for gestational age at birth (Figure 2).

**FIGURE 3:**
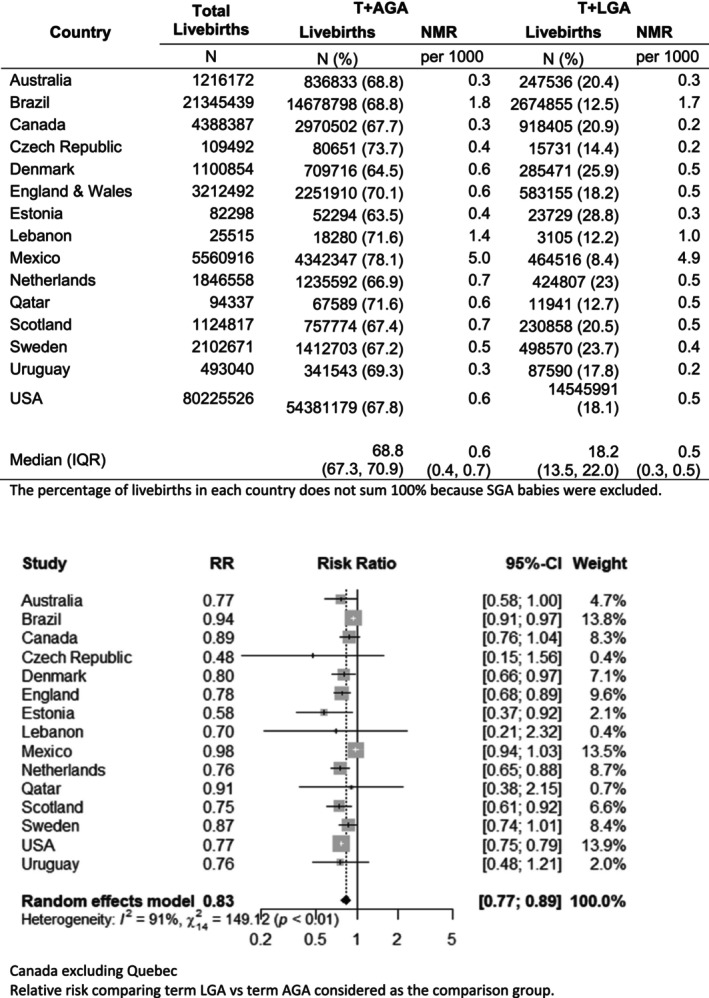
Number of live births, prevalence of newborn types (%), neonatal mortality rate (NMR) and pooled relative risks ratios (LGA vs AGA) in 15 countries, by size of the newborn.

Almost one‐fifth (21.0 million) live births were term + LGA (median prevalence, 18.2%; interquartile range, IQR, 13.5%–22.0%), and 84.1 million (median prevalence, 68.8%; IQR, 67.3%–70.9%) were term + AGA. The prevalence of term + LGA babies ranged from 8.4% in Mexico to 28.8% in Estonia. Neonatal mortality was lower in the LGA group (median NMR, 0.5 per 1000 live births; IQR, 0.3–0.5) compared with the AGA group (median NMR, 0.6 per 1000 live births; IQR, 0.4–0.7). Overall, term + LGA had a 17% lower neonatal mortality risk (RR 0.83, 95% CI 0.77–0.89) compared with term + AGA (Figure 3). The overall mortality risk of term LGA (vs AGA) showed little variation between early (RR 0.87, 95% CI 0.81–0.94) and late (RR 0.81, 95% CI 0.69–0.94) neonatal periods (Figure S3).

Among countries with data using the 95th and 97th centiles of LGA, the risk of neonatal mortality was higher for those born >95th centile from 39 weeks of gestation onwards in Brazil than their AGA counterparts. Whereas in Canada, an increased risk was only observed among post‐term babies born above the 97th centile (RR 3.39, 95% CI 1.02–11.2), and no increased mortality risk was observed for post‐term LGA babies born >97th centile in the USA or in Sweden (Table S6).

### 3.2 Prevalence and neonatal mortality associated with macrosomia

**FIGURE 4:**
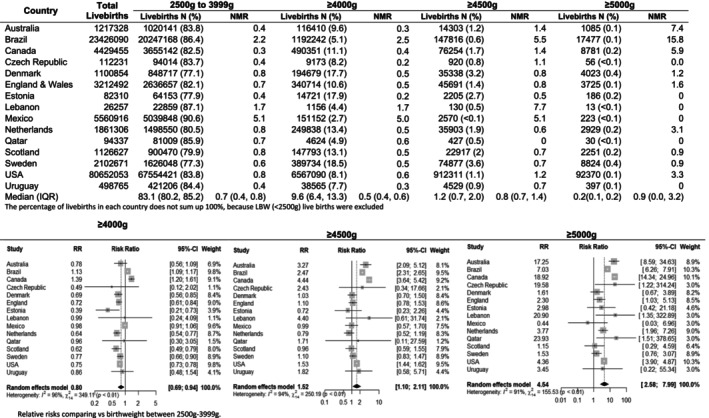
Number of livebirths, prevalence (%), neonatal mortality rate (NMR), and relative risks in 15 countries, by birthweight categories.

We identified 125.5 million live births and 576 018 deaths with birthweight recorded. Of these, 9.9 million live births were born weighing ≥4000 g (median prevalence, 9.6%; IQR, 6.4%–13.3%) and 105.7 million live births were born weighing 2500–3999 g (median prevalence, 83.1%; IQR, 80.2%–85.2%). Among macrosomic live births, 1.3 million had birthweights of ≥4500 g (median prevalence, 1.2%; IQR, 0.7%–2.0%) and 0.1 million had birthweights of ≥5000 g (median prevalence, 0.2%; IQR, 0.1%–0.2%). The prevalence of macrosomic live births was relatively higher in Sweden (18.5%), Estonia (17.9%) and Denmark (17.7%), and was relatively lower in Brazil (5.1%), Lebanon (4.4%), Qatar (4.9%) and Mexico (2.7%) (Figure 4).

**FIGURE 5:**
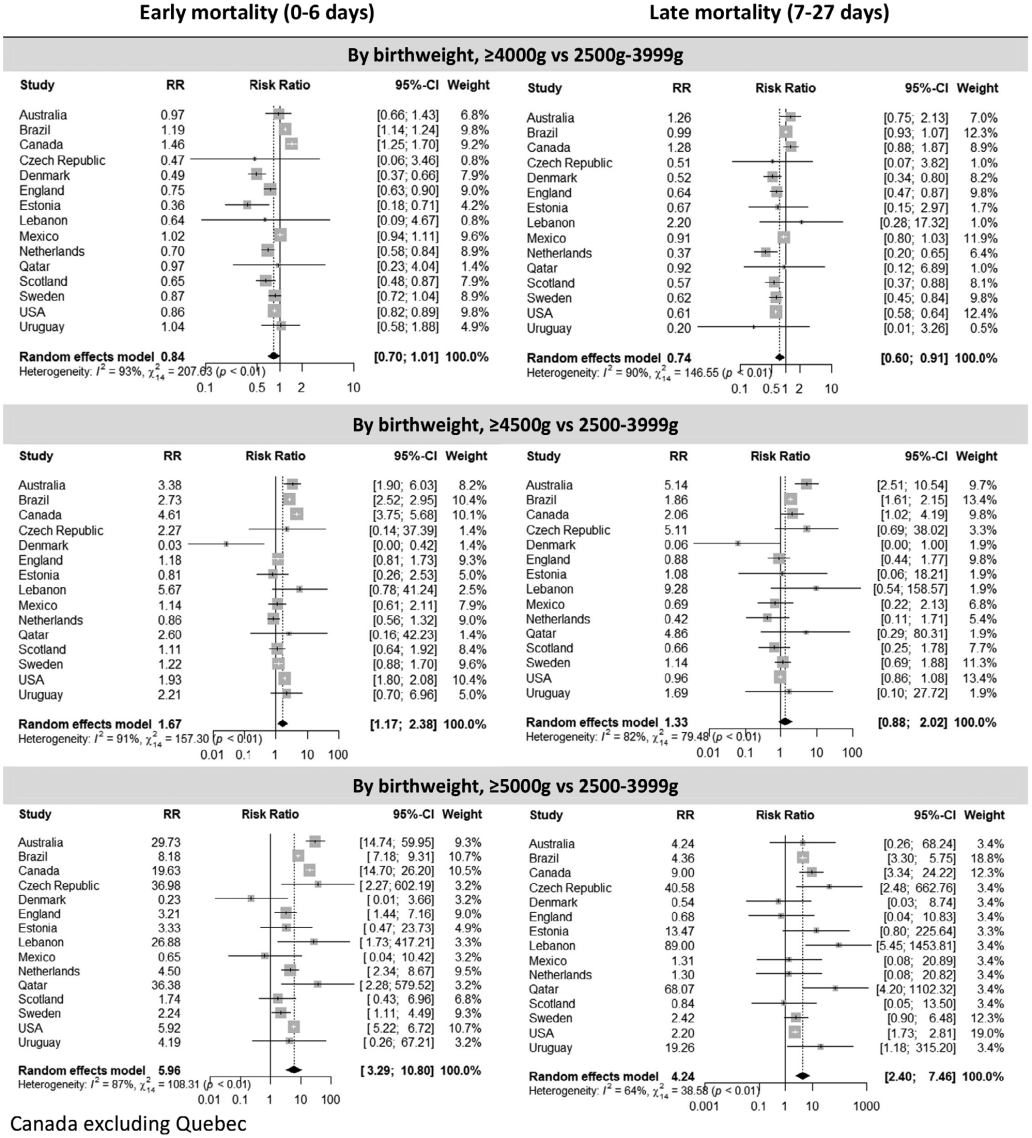
Forest plots summarising the relative risks of early and late neonatal mortality in macrosomic babies (≥4000, ≥4500 and ≥5000 g) versus babies born weighing 2500–3999 g.

The median mortality risk showed a gradient from 0.5 deaths per 1000 live births in the group born weighing ≥4000 g (IQR 0.4–0.6 deaths per 1000 live births) to 0.8 deaths per 1000 live births among those born weighing ≥4500 g (IQR 0.7–1.4 deaths per 1000 live births) and to one death per 1000 live births among those weighing ≥5000 g (IQR 0–3.2 deaths per 1000 live births). Overall, macrosomic live births weighing ≥4000 g had a lower risk of neonatal mortality than those with birthweights of 2500–3999 g (RR 0.80, 95% CI 0.69–0.94); however, the subset of babies born weighing ≥4500 g had an elevated risk (RR 1.52, 95% CI 1.10–2.11) (Figure 4). The highest risk for those born weighing ≥4500 g was observed in the early neonatal period (days 0–6). Babies born weighing ≥4500 g had a 67% increase in early neonatal mortality (RR 1.67, 95% CI 1.17–2.38), compared with 33% in the later period (days 7–27; RR 1.33, 95% CI 0.88–2.02). The greatest risk was seen in babies born weighing ≥5000 g (days 0–6, RR 5.96, 95% CI 3.29–10.80; days 7–27, RR 4.24, 95% CI 2.40–7.46) (Figure 5).

Five countries (Czech Republic, Lebanon, Mexico, Qatar and Uruguay) had no deaths in the group weighing ≥5000 g, but the RR was the highest for all countries with deaths in this group. For instance, live births weighing ≥5000 g in Australia (RR 17.25, 95% CI 8.59–34.63), Brazil (RR 7.03, 95% CI 6.26–7.91), Canada (RR 18.92, 95% CI 14.34–24.96) and the USA (RR 4.36, 95% CI 3.90–4.87) had the highest risk of neonatal mortality overall, followed by those born weighing ≥4500 g in Australia (RR 3.27, 95% CI 2.09–5.12), Brazil (RR 2.47, 95% CI 2.31–2.65), Canada (RR 4.44, 95% CI 3.64–5.42) and the USA (RR 1.53, 95% CI 1.44–1.62), compared with those born weighing 2500–3999 g, which are considered as the reference group. England & Wales (RR 2.30, 95% CI 1.03–5.13) and the Netherlands (RR 3.77, 95% CI 1.96–7.26) showed an increased risk of mortality among the group born weighing ≥5000 g but not among those born weighing ≥4500 g (Figure 4).

## 4 DISCUSSION

### 4.1 Main findings

Our data set of more than 115 million live births enabled a multi‐country analysis of LGA and macrosomia associated with neonatal mortality in 15 middle‐ and high‐income countries. LGA was common, affecting around 20% of births, but this group did not show a greater risk of neonatal mortality compared with AGA; in contrast, whereas overall macrosomia of ≥4000 g was not associated with increased neonatal mortality, birthweights of ≥4500 g were associated with higher mortality, with the highest increased risk for those born weighing ≥5000 g during the early neonatal period.

We observed substantial variation in the prevalence and mortality risk of macrosomia in the 15 national cohorts. Overall, the threshold of ≥4500 g was a useful marker for mortality risk, but not all countries showed an increased risk in this group. These national variations might reflect true differences in maternal risk factors (including, for instance, overweight, obesity and diabetes)^36^ as well as differences in care, including access to intensive obstetric monitoring and labour induction practices, delivery mode preferences and access to high‐quality postnatal care.^14^, ^25^, ^37^, ^38^ Given the variability in the neonatal mortality risk among the macrosomic group, we emphasise the value of conceptualising macrosomia considering a broader clinical picture throughout the continuum of care, rather than a single threshold, with respect to the clinical and prognostic value of birthweights of ≥4000 g.

Although LGA >90th centile was not associated with an increased risk of neonatal mortality, analysis by centiles and gestational age showed a higher risk of mortality among LGA babies over the 97th centile (vs AGA) born post‐term in Brazil and Canada, but not in the USA or Sweden. Of note, very few births or neonatal deaths occurred in these post‐term groups, probably because of active induction protocols for post‐term delivery in these settings. However, in Brazil, where there is greater variation in obstetric care practices, the increased neonatal risk of mortality for those born >95th centile from 39 weeks of gestation onwards requires additional investigation, to determine which potential modifiable factors could contribute to this risk and inform future clinical management pathways.

### 4.2 Interpretation

Our analysis adapted a recent classification of ‘vulnerable newborns’ to identify the most vulnerable babies at risk for neonatal mortality. Our research indicated that LGA does not reflect vulnerability in a newborn but helps to identify extreme thresholds of birthweight to predict early mortality. The elevated risk of fatal outcomes for neonates born with birthweights of 4500 g and above, or >97th centile, was generally in agreement with findings from previous studies.^14^, ^23^, ^39^, ^40^

We hypothesised that big babies were at the highest risk of death during the first week after birth, probably associated with birth trauma and subsequent asphyxia. Moreover, the mortality risk remained elevated in the late neonatal period (especially among those weighing 5000 g and above), probably because of delayed effects of intrapartum complications, hypoglycaemia, congenital anomalies and infection during prolonged hospitalisations.^41^, ^42^ Future applications of this analysis include a more granulated description of subgroups of macrosomia, a consideration of maternal risk factors and implications for the early detection, monitoring and medical care of metabolic conditions that can lead to hyperglycaemia, the excessive secretion of insulin, fat deposition and fetal organomegaly,^6^, ^43^, ^44^ such as overweight, obesity and gestational diabetes.^11^ The increase in obesity and diabetes among women of childbearing age in recent decades has potential public health importance, because of the consequent rise in the proportions of LGA or ‘macrosomic’ babies.^26^, ^45^

In clinical practice, the prenatal identification of maternal risk factors and large estimated fetal size may be beneficial to inform labour management decisions, including induction, planned delivery in higher‐level facilities with appropriate skilled personnel and reliable infrastructure to facilitate intrapartum monitoring and timely interventions, including caesarean section, when indicated.^31^, ^46^, ^47^, ^48^ Although we recognise the modest predictive performance and considerable costs to detect big babies using routine third‐trimester ultrasounds, in particular in low‐ and middle‐income settings, this may perform better by combining maternal features, first‐trimester parameters and fetal biomarkers.^8^, ^49^, ^50^ For clinicians, the recognition of increased risks associated with large size at birth can inform neonatal management, including monitoring for early detection and intervention for metabolic, neurological and respiratory complications.^41^, ^51^

### 4.3 Strengths and limitations

This multi‐country analysis including over 115 million live births and half a million neonatal deaths provided sample statistical power to examine relatively rare outcomes by using nationwide administrative data sets from 15 high‐ and middle‐income countries. The INTERGROWTH‐21st standards for gestational age and sex enabled international comparisons and exploration of the prevalence and mortality risks of LGA babies. We were able to examine various combinations of LGA and macrosomic thresholds to identify degrees of risk for neonatal mortality.

Although we were able to compile information on live births and neonatal deaths by using large administrative data sets, our findings pose a challenge for generalisability, because of the substantial variability to the expected 10% of LGA and the lack of data from low‐income countries.

In addition, we recognise that the proportion of missing values, for example birthweight (<0.1%–2.0%), gestational age (<0.1%–8.7%) and sex (<0.1%–2.5%), pose some limitations in calculating mortality risks, and there is also variation in the completeness of the linkages between live births and neonatal deaths across countries (Tables S4 and S5).^52^, ^53^

In terms of mortality risk, we were unable to adjust for potential confounding factors, such as maternal age, ethnicity, overweight, obesity and diabetes, which affects the interpretation of our results.^54^ Our analysis examined neonatal mortality in large liveborn infants and should be interpreted with consideration, and further analysis including stillbirths would be valuable to explore perinatal mortality overall.^46^

## 5 CONCLUSION

This study revealed a high prevalence of babies born LGA in high‐income countries, who have a relatively lower risk of neonatal mortality than those born as AGA infants. In contrast, macrosomia was less common but was a stronger risk factor for neonatal mortality, particularly during the first week. These findings are valuable to guide clinical management, monitoring and information for parents.

## AUTHOR CONTRIBUTIONS

The Vulnerable Newborn Measurement Collaboration was planned by JEL and REB. This analysis was designed by HB and EOO with JEL. Analysis was undertaken by LS‐I with EOO. A systematic review of LGA babies was conducted by C‐JC, supervised by HB. AG, JS, HLSL, ESP, IRF, SL, QW, PV, JJ, EH‐P, HTS, LS, LA, KAY, AAB, SLA, LB, AEvD, FA, MA, NR, JS, LKS, RJM, EL, NR, RW, KM, IP and GP analysed and verified national data outputs. The article was drafted by LSI, together with HB, EOO and JEL. EAH, JY, YBO and EB provided inputs and helped revise the article. All authors contributed to the study protocol, which included input from the wider Vulnerable Newborn Measurement Collaboration. All authors reviewed and agreed on the final version.

## CONFLICT OF INTEREST STATEMENT

No competing interests to declare.

## FUNDING INFORMATION

This work received funding from the Children's Investment Fund Foundation (prime grant 1803‐02535). The funders had no role in the study design, data collection, analysis or interpretation for the article.

## ETHICS APPROVAL

The Vulnerable Newborn Measurement Collaboration was granted ethical approval from the Institutional Review Boards of the London School of Hygiene & Tropical Medicine (ref. 22858) and Johns Hopkins Bloomberg School of Public Health (IRB no. 16439, 8 May 2021). All 15 country teams were granted ethical approval for the use of data or exemptions according to the current remit.

## AUSTRALIAN DATA DISCLOSURE

We are grateful to the Consultative Council on Obstetric and Paediatric Mortality and Morbidity (CCOPMM) for providing access to the data used in this project and to the staff at Safer Care Victoria for providing assistance. The conclusions, findings, opinions and views or recommendations expressed herein are strictly those of the authors and do not necessarily reflect those of CCOPMM. We acknowledge and thank the NT Perinatal Data team for access to the Northern Territory perinatal data collection. Australian data were provided to the CRE team and international team with small numbers, i.e. those less than five, suppressed.

## Supporting information


Appendix S1.

## Data Availability

Data sharing and transfer agreements were jointly developed and signed by all collaborating partners. The pooled aggregate data will be available at https://doi.org/10.17037/DATA.00003095 at the time of publication, with the exception of data from countries where data sharing is not permitted.

## APPENDIX 1

### 1.1

National Collaborative Group for Vulnerable Newborn Mortality members: Australia: Vicki Flenady, Adrienne Gordon, Kara Warrilow, Harriet Lawford and Jessica Sexton. Brazil: Enny S. Paixao, Mauricio Lima Barreto and Ila Rocha Falcão. Canada: Sarka Lisonkova and Qi Wen. Czech Republic: Petr Velebil and Jitka Jírová. Denmark: Erzsébet Horváth‐Puhó and Henrik T. Sørensen. Estonia: Luule Sakkeus and Liili Abuladze. Lebanon: Khalid A. Yunis, Ayah Al Bizri and Pascale Nakad. Mexico: Lorena Suárez‐Idueta, Arturo Barranco Flores, Jesus Felipe Gonzalez Roldan and Sonia Lopez Alvarez. The Netherlands: Lisa Broeders and Aimée E. van Dijk. Qatar: Fawzia Alyafei, Mai AlQubaisi, Tawa O. Olukade, Hamdy A. Ali and Mohamad Rami Alturk. Sweden: Neda Razaz and Jonas Söderling. UK: England and Wales: Lucy K. Smith, Bradley N. Manktelow, Ruth J. Matthews, Elizabeth Draper, Alan Fenton and Jennifer J. Kurinczuk. UK: Northern Ireland: Estelle Lowry and Neil Rowland. UK: Scotland: Rachael Wood, Celina Davis, Kirsten Monteath and Samantha Clarke. Uruguay: Isabel Pereyra and Gabriella Pravia. USA: Sarka Lisonkova and Qi Wen.

#### Vulnerable Newborn Measurement Core Group

LSHTM: Joy E. Lawn, Hannah Blencowe, Eric O. Ohuma, Yemisrach B. Okwaraji, Judith Yargawa and Ellen Bradley. JHU: Robert E. Black, Joanne Katz, Daniel Erchick, Elizabeth A. Hazel, Michael Diaz and Anne C. C. Lee.
