# Supplementary material for: Neonatal mortality risk of large‐for‐gestational‐age and macrosomic live births in 15 countries, including 115.6 million nationwide linked records, 2000–2020
Source: BJOG. 2023 Nov 27;132(Suppl 8):S109–20. doi: 10.1111/1471-0528.17706 (PMC12678065; doi:10.1111/1471-0528.17706)
Supplement: Supplementary file 1 — Appendix S1. [file BJO-132-S109-s001.docx]

**SUPPLEMENT TITLE**: Vulnerable Newborn multi-country analyses related to preterm births and small-for-gestational age

**PAPER TITLE**

Neonatal mortality risk for large-for-gestational age and macrosomic livebirths in 15 countries including 115.6 million nationwide linked records, 2000 to 2020

**PAPER RUNNING TITLE**

Mortality risk for LGA and macrosomia in 15 countries

**SUPPORTING INFORMATION**

Table of Contents

[**Table S1: Definitions** 2](#_Toc145871235)

[**Table S2: RECORD guidelines** 3](#_Toc145871236)

[**Table S3: Ethics approval or exemptions of Institutional Review Boards** 10](#_Toc145871237)

[**Input data** 11](#_Toc145871238)

[**Table S4. Data quality assessment. Number of missing values on three core variables (birthweight, gestational age, and sex) in 15 countries** 11](#_Toc145871239)

[**Table S5. Summary of metadata** 12](#_Toc145871240)

[**Figure S1a. Methods used to extend INTERGROWTH-21st newborn size standards from 22^+0^ to 44^+6^** 14](#_Toc145871241)

[**Figure S1b. Birthweight centiles used from 37 to 42 weeks** 14](#_Toc145871242)

[**Figure S2. Overview of vulnerable newborn types categorised by gestational age, size for gestational age and birthweight.** 15](#_Toc145871243)

[**Figure S3. Forest plots of summary statistics derived from random effects model for early and late neonatal mortality risks in LGA babies (vs AGA)** 16](#_Toc145871244)

[**Table S6: Number of livebirths, deaths, and relative risk of neonatal mortality (vs AGA) by LGA centiles (>90th , >95th and >97 centiles) , in Brazil, Canada, Sweden and the US** 17](#_Toc145871245)

[**Additional references** 18](#_Toc145871246)

# **Table S1: Definitions**

Consistent with the International Classification of Disease 10th and 11^th^ revisions ^1, 2^. INTERGROWTH-21st international standards ^3, 4^

| **Definitions** |  |
| --- | --- |
| Livebirth | Is the complete expulsion or extraction from a woman of a fetus, irrespective of the duration of the pregnancy, which, after such separation, shows signs of life |
| Birthweight | Is defined as the weight of the fetus or newborn obtained immediately after birth. For livebirths, measurement of birthweight within the first hour of life before significant postnatal weight loss has occurred is preferable. If the birth weight was measured repeatedly, the median value will be used. Birthweight can be measured using digital or analogue scales. For the purposes of this work, weights of newborn taken at ≥72 hours after birth will be excluded. |
| Gestational age | The duration of gestation measured from the first day of the last menstrual period (LMP). Gestational age will be analysed in days where possible. Gestational age measured by LMP, early pregnancy ultrasound or best obstetric estimate (BEO) will be included |
| Neonatal death | A neonatal death is defined as a death during the first 28 days after live birth (days 0-27). An early neonatal death is a death during the first 7 days after live birth (days 0 – 6), a late neonatal death is a death day 7 – 27 after a livebirth. |
| **Calculated variables** | |
| Preterm birth | A birth before 37 completed weeks of gestation (or before 259 days of gestation) as measured from the first day of the last menstrual period (LMP) or by early ultrasound. |
| Term birth | A birth from 37 completed weeks of gestation as measured from the first day of the last menstrual period (LMP) or by early ultrasound. |
| Small for gestational age | Birth with a birthweight for gestational age and sex of <10th centile according to INTERGROWTH-21st international standards |
| Appropriate for gestational age | A birth with a birthweight for gestational age and sex from 10th to 90th centiles according to INTERGROWTH-21st international standards |
| Large for gestational age | Birth with a birthweight for gestational age and sex of >90th centile according to INTERGROWTH-21st international standards |
| Low birthweight | A birth with a birthweight below 2500g |
| Macrosomia | A birth with a birthweight ≥4000g regardless of any gestational age assessment |

# **Table S2: RECORD guidelines**

**The reporting of studies conducted using observational routinely collected data (RECORD) checklist**

|  | **#** | **STROBE items** | **Location** | **RECORD items** | **Location in manuscript where items are reported** |
| --- | --- | --- | --- | --- | --- |
| **Title and abstract** | | | | | |
|  | 1 | (a) Indicate the study’s design with a commonly used term in the title or the abstract (b) Provide in the abstract an informative and balanced summary of what was done and what was found |  | RECORD 1.1: The type of data used should be specified in the title or abstract. When possible, the name of the databases used should be included.  RECORD 1.2: If applicable, the geographic region and timeframe within which the study took place should be reported in the title or abstract.  RECORD 1.3: If linkage between databases was conducted for the study, this should be clearly stated in the title or abstract. | Neonatal mortality risk for large-for-gestational age and macrosomic livebirths in 15 countries including 115.6 million nationwide linked records, 2000 to 2020 |
|  | | | | | |
| Background rationale | 2 | Explain the scientific background and rationale for the investigation being reported |  |  | Introduction  (Paragraphs 1-4) |
| Objectives | 3 | State specific objectives, including any prespecified hypotheses |  |  | Introduction  (Paragraph 4) |
|  | | | | | |
| Study Design | 4 | Present key elements of study design early in the paper |  |  | Methods  (Paragraph 1-3) |
| Setting | 5 | Describe the setting, locations, and relevant dates, including periods of recruitment, exposure, follow-up, and data collection |  |  | Methods  (Paragraphs 2-3) |
| Participants | 6 | *(a) Cohort study* - Give the eligibility criteria, and the sources and methods of selection of participants. Describe methods of follow-up  *Case-control study* - Give the eligibility criteria, and the sources and methods of case ascertainment and control selection. Give the rationale for the choice of cases and controls  *Cross-sectional study* - Give the eligibility criteria, and the sources and methods of selection of participants  *(b) Cohort study* - For matched studies, give matching criteria and number of exposed and unexposed  *Case-control study* - For matched studies, give matching criteria and the number of controls per case |  | RECORD 6.1: The methods of study population selection (such as codes or algorithms used to identify subjects) should be listed in detail. If this is not possible, an explanation should be provided.  RECORD 6.2: Any validation studies of the codes or algorithms used to select the population should be referenced. If validation was conducted for this study and not published elsewhere, detailed methods and results should be provided.  RECORD 6.3: If the study involved linkage of databases, consider use of a flow diagram or other graphical display to demonstNMR the data linkage process, including the number of individuals with linked data at each stage. | Methods  (Paragraphs 4-5)  Under the subheading Data quality, inclusion and Exclusion criteria  Figure 3. Flowchart |
| Variables | 7 | Clearly define all outcomes, exposures, predictors, potential confounders, and effect modifiers. Give diagnostic criteria, if applicable. |  | RECORD 7.1: A complete list of codes and algorithms used to classify exposures, outcomes, confounders, and effect modifiers should be provided. If these cannot be reported, an explanation should be provided. | Methods  (Paragraphs 8-9) under the subheading exposure definitions |
| Data sources/ measurement | 8 | For each variable of interest, give sources of data and details of methods of assessment (measurement).  Describe comparability of assessment methods if there is more than one group |  |  | Methods  (Paragraphs 8-9) under the subheading exposure definitions |
| Bias | 9 | Describe any efforts to address potential sources of bias |  |  | Quality assessment described in Methods (Paragraph 5 and Table S4) |
| Study size | 10 | Explain how the study size was arrived at |  |  |  |
| Quantitative variables | 11 | Explain how quantitative variables were handled in the analyses. If applicable, describe which groupings were chosen, and why |  |  | Methods (Paragraphs 7-8) |
| Statistical methods | 12 | (a) Describe all statistical methods, including those used to control for confounding  (b) Describe any methods used to examine subgroups and interactions  (c) Explain how missing data were addressed  (d) *Cohort study* - If applicable, explain how loss to follow-up was addressed  *Case-control study* - If applicable, explain how matching of cases and controls was addressed  *Cross-sectional study* - If applicable, describe analytical methods taking account of sampling stNMRgy  (e) Describe any sensitivity analyses |  |  | Methods (Paragraph 10-11) under the subheading analysis |
| Data access and cleaning methods |  | .. |  | RECORD 12.1: Authors should describe the extent to which the investigators had access to the database population used to create the study population.  RECORD 12.2: Authors should provide information on the data cleaning methods used in the study. | Methods (Paragraph 5-6) |
| Linkage |  | .. |  | RECORD 12.3: State whether the study included person-level, institutional-level, or other data linkage across two or more databases. The methods of linkage and methods of linkage quality evaluation should be provided. | Supplementary table S4b Summary of metadata |
| **Results** | | | | | |
| Participants | 13 | (a) Report the numbers of individuals at each stage of the study (*e.g.*, numbers potentially eligible, examined for eligibility, confirmed eligible, included in the study, completing follow-up, and analysed)  (b) Give reasons for non-participation at each stage.  (c) Consider use of a flow diagram |  | RECORD 13.1: Describe in detail the selection of the persons included in the study (*i.e.,* study population selection) including filtering based on data quality, data availability and linkage. The selection of included persons can be described in the text and/or by means of the study flow diagram. | Results (Paragraph 2-4 and Figure 3) |
| Descriptive data | 14 | (a) Give characteristics of study participants (*e.g.*, demographic, clinical, social) and information on exposures and potential confounders  (b) Indicate the number of participants with missing data for each variable of interest  (c) *Cohort study* - summarise follow-up time (*e.g.*, average and total amount) |  |  | Results paragraphs 1-4 |
| Outcome data | 15 | *Cohort study* - Report numbers of outcome events or summary measures over time  *Case-control study* - Report numbers in each exposure category, or summary measures of exposure  *Cross-sectional study* - Report numbers of outcome events or summary measures |  |  | Results (Paragraph 2-6, Tables 1-3) |
| Main results | 16 | (a) Give unadjusted estimates and, if applicable, confounder-adjusted estimates and their precision (e.g., 95% confidence interval). Make clear which confounders were adjusted for and why they were included  (b) Report category boundaries when continuous variables were categorized  (c) If relevant, consider translating estimates of relative risk into absolute risk for a meaningful time period |  |  | Results (Paragraph 2-6, Tables 1-3) |
| Other analyses | 17 | Report other analyses done—e.g., analyses of subgroups and interactions, and sensitivity analyses |  |  | Results (Paragraph 3, supplementary tables S5 and S6) |
|  | | | | | |
| Key results | 18 | Summarise key results with reference to study objectives |  |  | Discussion under the subheading main findings and Figure 1 |
| Limitations | 19 | Discuss limitations of the study, taking into account sources of potential bias or imprecision. Discuss both direction and magnitude of any potential bias |  | RECORD 19.1: Discuss the implications of using data that were not created or collected to answer the specific research question(s). Include discussion of misclassification bias, unmeasured confounding, missing data, and changing eligibility over time, as they pertain to the study being reported. | Discussion (Paragraphs 5) under the subheading strengths and limitations |
| Interpretation | 20 | Give a cautious overall interpretation of results considering objectives, limitations, multiplicity of analyses, results from similar studies, and other relevant evidence |  |  | Discussion (Paragraphs 3) under the subheading interpretation |
| Generalisability | 21 | Discuss the generalisability (external validity) of the study results |  |  | Discussion (Paragraphs 5) under the subheading strengths and limitations |
|  | | | | | |
| Funding | 22 | Give the source of funding and the role of the funders for the present study and, if applicable, for the original study on which the present article is based |  |  | The source of founding is included in the Abstract and the funding role is described at the end of the manuscript under the subheading Funding role |
| Accessibility of protocol, raw data, and programming code |  | .. |  | RECORD 22.1: Authors should provide information on how to access any supplemental information such as the study protocol, raw data, or programming code. | Under the subheading Data availability statement |

Source ^5^

# **Table S3: Ethics approval or exemptions of Institutional Review Boards**

| **Country of origin for data** | **Institutional Review Board(s) or data access provider** | **Ref/Number** | **Date of approval** |
| --- | --- | --- | --- |
| London School of Hygiene & Tropical Medicine (LSHTM) | LSHTM - Observational / Interventions Research Ethics Committee | 22858 | 17^th^ May 2021 |
| Australia | Australian Institute of Health and Welfare Ethics Committee | EO2018/2/451 | 4th May 2021 |
| Brazil | Federal University of Bahia’s Institute of Public Health Ethics Committee | 18022319.4.0000.5030 | 3^rd^ September 2019 |
| Canada | UBC C&W Research Ethics Board | H21-00653 | 31^st^ March 2021 |
| Estonia | Ethics Committee of National Institute for Health Development | 770 | 9^th^ August 2021 |
| Lebanon | Institutional Review Board, American University of Beirut | PED.KY.01 | 13^th^ July 2021 |
| Mexico | Centre of Investigation in Health Sciences, Anahuac University, Mexico | 202214 | 31^st^ March 2022 |
| Qatar | Medical Research Center, Hamad Medical Corporation, Doha-Qatar | MRC-01-21-277 | 25^th^ April 2021 |
| England and Wales | 1. National Information Governance Board  2. Confidentiality Advisory Group of the Health Research Authority  3. Health & Social Care Information Centre (HSCIC), Data Access Advisory Group | 1. ECC 5-05 (f)/2012  2. 15/CAG/0119  3. DARS-NIC-359651-H3R1P-v5.2. | 10^th^ October 2012 and  1^st^ May2015 |
| Scotland | Public Health Scotland | 20210218-VulnerableNewbornMeasurement | 30^th^ March 2021 |
|  | | | |
| **Exemptions (*e.g., IRB approval not required for public or aggregate data, existing ethics approval in place, etc)*** | | | |
| Czech Republic | | | |
| Denmark | | | |
| Netherlands | | | |
| Sweden | | | |
| Uruguay | | | |
| USA publicly available data from https://www.cdc.gov/nchs/data_access/Vitalstatsonline.htm | | | |

# **Input data**

# **Table S4. Data quality assessment. Number of missing values on three core variables (birthweight, gestational age, and sex) in 15 countries**

| **Country** | **Period of observation** | **Livebirths** | **Missing values** | | | | | |
| --- | --- | --- | --- | --- | --- | --- | --- | --- |
|  |  |  | **Birthweight** | | **Gestational age** | | **Sex** | |
|  | **(years)** | **(n)** | **(n)** | **(%)** | **(n)** | **(%)** | **(n)** | **(%)** |
| Australia | 2016-2019 | 1,217,919 | 539 | <0.1 | 548 | <0.1 | 108 | <0.1 |
| Brazil | 2011-2018 | 23,439,789 | 13,442 | 0.1 | 2,036,695 | 8.7 | 4,217 | <0.1 |
| Canada* | 2005-2020 | 4,163,541 | 347 | <0.1 | 34,794 | 0.8 | 160 | <0.1 |
| Czech Republic | 2019 | 112,231 | 1,512 | 1.3 | 2,719 | 2.4 | 0 | 0.0 |
| Denmark | 2000-2017 | 1,125,560 | 20,903 | 1.9 | 18,748 | 1.7 | 294 | <0.1 |
| England & Wales | 2015-2019 | 3,212,492 | 0 | 0.0 | 0 | 0.0 | 0 | 0.0 |
| Estonia | 2015-2020 | 82,427 | 0 | 0.0 | 0 | 0.0 | 0 | 0.0 |
| Lebanon | 2001 & 2017 | 26,792 | 535 | 2.0 | 709 | 2.6 | 677 | 2.5 |
| Netherlands | 2010-2019 | 1,861,400 | 2,277 | 0.1 | 12,520 | 0.7 | 59 | <0.1 |
| Qatar | 2016-2019 | 95,906 | 350 | 0.4 | 1,202 | 1.3 | 5 | <0.1 |
| Scotland | 2000-2020 | 1,127,984 | 1,357 | 0.1 | 1,659 | 0.1 | 181 | <0.1 |
| Sweden | 2000-2019 | 2,102,671 | 0 | 0.0 | 0 | 0.0 | 0 | 0.0 |
| Uruguay | 2009-2020 | 499,345 | 580 | 0.1 | 5,350 | 1.1 | 0 | 0.0 |
| US | 2000-2019 | 80,710,348 | 78,490 | 0.1 | 317,158 | 0.4 | 1,672 | <0.1 |

*Excluding Quebec

# **Table S5. Summary of metadata**

| **Country** | **Units for reporting**  **Birthweight/ Gestational age** | **Data linkage** | **Reporting criteria for very preterm** | | |
| --- | --- | --- | --- | --- | --- |
|  |  |  | **Exclusions criteria based on BW** | **Exclusion criteria based on GA** | **Are births following induced Termination of Pregnancy included in the data source?** |
| Australia | Grams | Not applicable as livebirths, stillbirths and neonatal deaths are all included as part of the National Perinatal Data Collection | A small number of births <400 grams are included | A small number of births < 20 weeks are included | Both livebirths and stillbirths may include termination of pregnancy after 20 weeks. |
|  | Completed weeks |  |  |  |  |
| Brazil | Grams | Livebirths and death records were linked using the name of the mother, maternal date of birth or age (when the date of birth was missing), and the municipality of residence of the mother as matching variables using CIDACS-RL software | <350g | <20 weeks | No |
|  | Completed weeks |  |  |  |  |
| Canada | Grams | Data were obtained from the Discharge Abstract Database (DAD) which collates all hospitalization records of maternal hospitalizations for childbirth and also links maternal and live birth infant records. The province of Quebec does not contribute data to DAD. Home births were not included | None | None | No |
|  | Completed weeks |  |  |  |  |
| Czech Republic | Grams | Information system of newborn is linked at the individual level to the Registry of Reproductive Health and Information system of the dead | None | <22 weeks | No |
|  | Weeks +days |  |  |  |  |
| Denmark | Grams | Information on livebirths and stillbirths were extracted from the Danish Medical Birth Registry. These data were linked to the Danish Civil Registration System to define infant death | None | None | No |
|  | Days |  |  |  |  |
| England & Wales | Grams | Data linkage with birth notifications, and birth and stillbirth registrations and neonatal death registrations | None | 22 weeks | No |
|  | Completed weeks |  |  |  |  |
| Estonia | Grams | Estonian Medical Birth Register is Linked at the individual level to the Registry of Causes of Death | None | <22 weeks | No |
|  | Weeks +days |  |  |  |  |
| Lebanon | Grams  Weeks+ days |  | None | <22 weeks | No |
| Mexico | Weeks +days | Livebirths and deaths records were linked using the variables sex, date of birth, place of residence and place of occurrence using CIDACS-RL software | None | None | No |
|  | Completed weeks |  |  |  |  |
| Netherlands | Grams  Weeks + days | Records from midwifes, clinical obstetricians/gynaecologists and neonatologist are linked nationally | Gestational age ≥22 weeks; if g.a. missing, birthweight ≥500g | Gestational age ≥22 weeks; if g.a. missing, birthweight ≥500g | Both livebirths and stillbirths up to 24 weeks gestation may include termination of pregnancy |
| Qatar | Kilograms and grams  Weeks and days | Not applicable | None | None | No |
| Scotland | Grams | SMR02 is an episode based patient record relating to inpatients and day cases discharged from Obstetric specialties. NRS infant deaths were linked to the NRS livebirths (National Records of Scotland is responsible for registering births and deaths) to find the mother unique personal identifier, which was then used to link the death to the SMR02 record. | None | None | No |
|  | Completed weeks |  |  |  |  |
| Sweden | Grams | National databases that are linked together using the person-unique national registration numbers of children | None | <23 weeks | Included after 22 weeks |
|  | Days |  |  |  |  |
| Uruguay | Grams | Information on livebirths and stillbirths were extracted from the ﻿Uruguay Perinatal Computer Systemdata. These data were linked to the Uruguay Registry of Causes of Death to define infant death | None | None | No |
|  | Completed weeks |  |  |  |  |
| United States of America | Grams Completed weeks | Data on live births are available from the NCHS – Vital Statistics online. It includes data from live birth certificates. Linkages are performed to obtain information on infant death from Vital Statistics; the proportion of linked infant deaths is generally high, it differs by year (overall 98.7%-99.4%), and by state (e.g., 92.8%-100% in 2000) | None | None | The NCHS recommendation for fetal death definition is to exclude TOPs. Some states do exclude TOPs regardless of gestational age, while some states include TOPs |

#

# **Figure S1a. Methods used to extend INTERGROWTH-21st newborn size standards from 22^+0^ to 44^+6^**

For the purpose of this analysis, the published INTERGROWTH-21^st^ standards were extrapolated from the original models that covered the period from 24^+0^ to 42^+6^ by extending the range of gestational age starting from 22^+0^ to 23^+6^ weeks and from 43^+0^ to 44^+6^


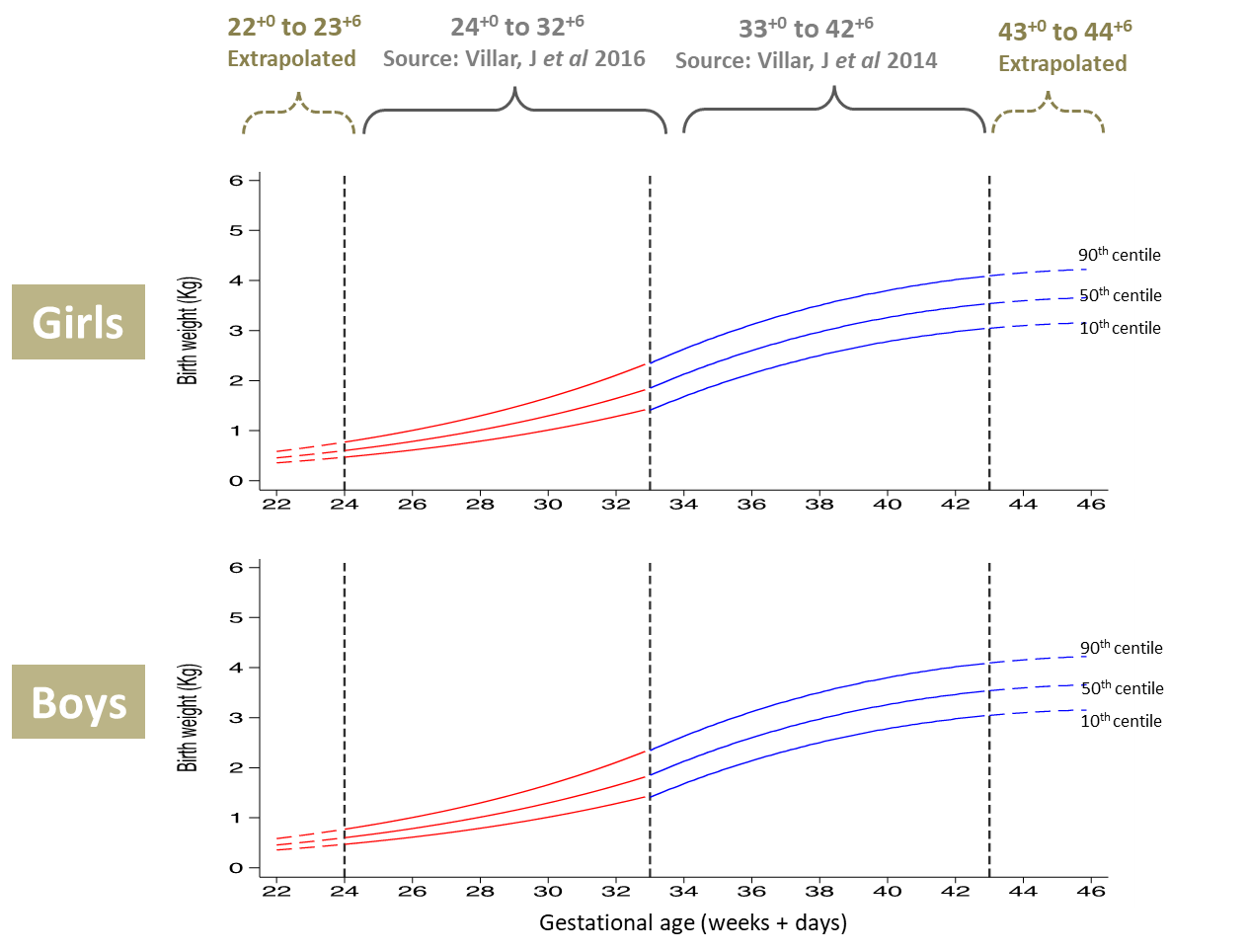


# **Figure S1b. Birthweight centiles used from 37 to 42 weeks**

| Weeks | Intergrowth 21st- Birthweight centiles | | | | | | | | |
| --- | --- | --- | --- | --- | --- | --- | --- | --- | --- |
|  | Girls | | | | Boys | | | | |
|  | 10th | 90th | 95th | 97th | 10th | 90th | 95th | 97th |  |
| 37 | 2.41 | 3.40 | 3.57 | 3.68 | 2.47 | 3.53 | 3.70 | 3.82 |  |
| 38 | 2.57 | 3.58 | 3.74 | 3.85 | 2.64 | 3.70 | 3.87 | 3.99 |  |
| 39 | 2.71 | 3.72 | 3.89 | 4.01 | 2.80 | 3.86 | 4.03 | 4.15 |  |
| 40 | 2.83 | 3.85 | 4.02 | 4.14 | 2.94 | 3.99 | 4.16 | 4.28 |  |
| 41 | 2.93 | 3.96 | 4.13 | 4.25 | 3.06 | 4.11 | 4.28 | 4.40 |  |
| 42 | 3.01 | 4.05 | 4.22 | 4.34 | 3.17 | 4.21 | 4.39 | 4.50 |  |
|  |  |  |  |  |  |  |  |  |  |
|  |  | AGA |  |  |  |  |  |  |  |
|  |  | LGA |  |  |  |  |  |  |  |
|  |  | Macrosomia >4000 | | |  |  |  |  |  |
|  |  | Macrosomia ≥4500 | | |  |  |  |  |  |

Birthweight centiles at mid-week (e.g., 37+3, 38+3)

Sources: ^3, 4, 6^

# **Figure S2. Overview of vulnerable newborn types categorised by gestational age, size for gestational age and birthweight.**


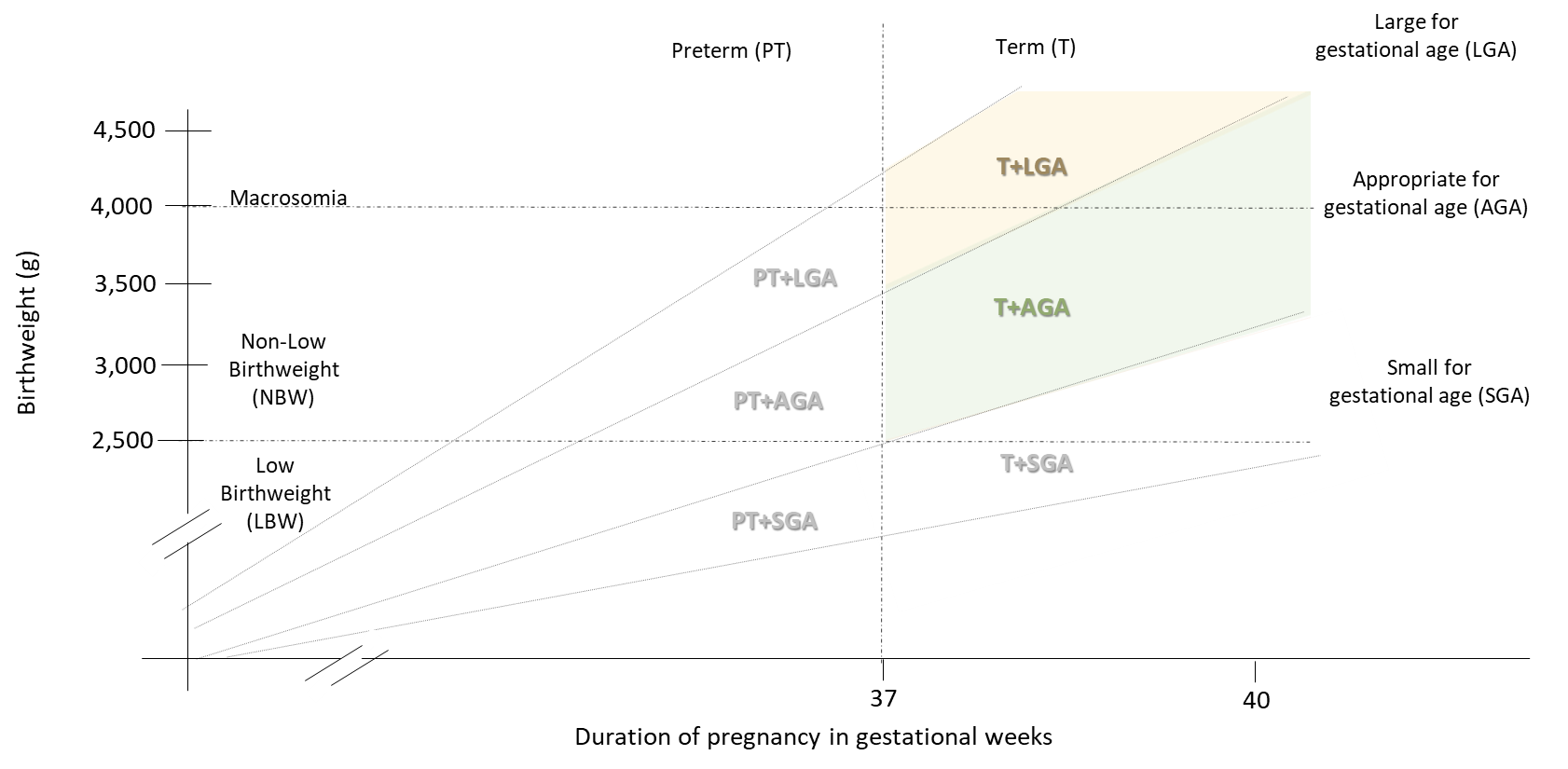


Focus on two newborn types included in this analysis to assess larger babies: term + LGA (in yellow) and term + AGA (in green).

**Additional outputs**

# **Figure S3. Forest plots of summary statistics derived from random effects model for early and late neonatal mortality risks in LGA babies (vs AGA)**

| **Early (0-6 days) neonatal mortality** | **Late (7-27 days) neonatal mortality** |
| --- | --- |
| **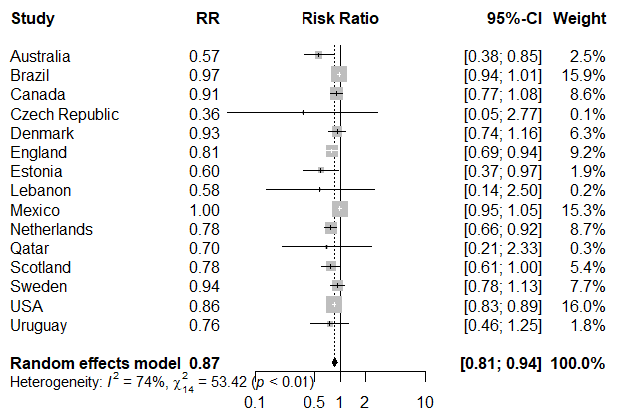** | **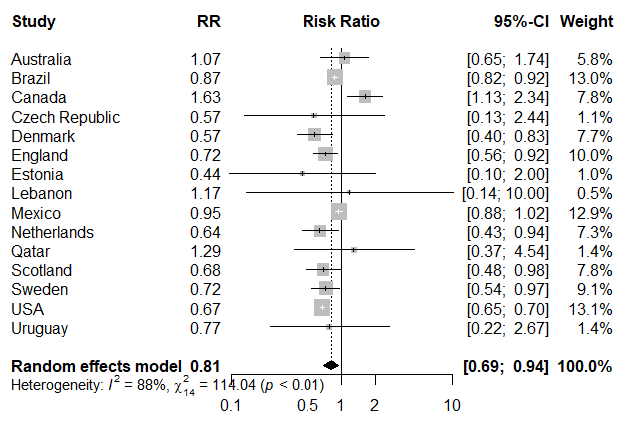** |

Canada excluding Quebec

Comparison group: term + AGA

# **Table S6: Number of livebirths, deaths, and relative risk of neonatal mortality (vs AGA) by LGA centiles (>90th , >95th and >97 centiles) , in Brazil, Canada, Sweden and the US**

| **Gestational age** | **AGA** | | **LGA (>90th centile)** | | | | **LGA (>95th centile)** | | | | **LGA (>97th centile)** | | | |
| --- | --- | --- | --- | --- | --- | --- | --- | --- | --- | --- | --- | --- | --- | --- |
|  | **Livebirths** | **Deaths** | **Livebirths** | **Deaths** | **RR** | **95%CI** | **Livebirths** | **Deaths** | **RR** | **95%CI** | **Livebirths** | **Deaths** | **RR** | **95%CI** |
| Brazil | | | | | | | | | | | | | | |
| 37 | 1,438,113 | 4,423 | 397,617 | 965 | 0.79 | (0.73,0.84) | 236,266 | 615 | 0.85 | (0.77,0.92) | 158,733 | 472 | 0.97 | (0.87,1.06) |
| 38 | 3,493,138 | 6,325 | 751,182 | 1,158 | 0.85 | (0.79,0.90) | 414,912 | 709 | 0.94 | (0.87,1.01) | 266,229 | 530 | 1.10 | (1.00,1.20) |
| 39 | 4,809,805 | 7,219 | 839,480 | 1,213 | 0.96 | (0.90,1.02) | 436,934 | 733 | 1.12 | (1.03,1.20) | 272,206 | 514 | 1.26 | (1.15,1.37) |
| 40 | 3,228,587 | 5,432 | 496,322 | 791 | 0.95 | (0.87,1.02) | 258,671 | 500 | 1.15 | (1.04,1.25) | 164,429 | 338 | 1.22 | (1.09,1.36) |
| 41 | 1,252,781 | 2,505 | 155,040 | 321 | 1.04 | (0.92,1.16) | 80,951 | 191 | 1.18 | (1.01,1.36) | 49,430 | 128 | 1.30 | (1.08,1.54) |
| >=42 | 456,825 | 1,129 | 33,672 | 119 | 1.43 | (1.18,1.72) | 13,550 | 58 | 1.73 | (1.33,2.25) | 8,177 | 47 | 2.33 | (1.73,3.11) |
| Canada | | | | | | | | | | | | | | |
| 37 | 253,308 | 124 | 66,621 | 43 | 1.32 | (0.93,1.86) | 40,431 | 24 | 1.21 | (0.78,1.87) | 28,322 | 20 | 1.44 | (0.89,2.31) |
| 38 | 606,881 | 190 | 191,186 | 42 | 0.70 | (0.50,0.98) | 115,822 | 26 | 0.72 | (0.47,1.08) | 78,850 | 21 | 0.85 | (0.54,1.33) |
| 39 | 893,845 | 164 | 272,836 | 34 | 0.68 | (0.46,0.98) | 157,836 | 24 | 0.83 | (0.54,1.27) | 104,946 | 18 | 0.93 | (0.57,1.52) |
| 40 | 830,337 | 148 | 259,501 | 47 | 1.02 | (0.73,1.41) | 150,806 | 23 | 0.86 | (0.55,1.32) | 101,266 | 15 | 0.83 | (0.48,1.41) |
| 41 | 374,954 | 86 | 123,900 | 23 | 0.81 | (0.51,1.28) | 73,232 | 15 | 0.89 | (0.51,1.54) | 49,014 | 11 | 0.98 | (0.52,1.83) |
| >=42 | 11,135 | 8 | 4,098 | 8 | 2.72 | (1.02,7.23) | 2,437 | 4 | 2.28 | (0.68,7.58) | 1,643 | 4 | 3.39 | (1.02,11.2) |
| US | | | | | | | | | | | | | | |
| 37 | 5,274,467 | 7,080 | 1,336,339 | 1,473 | 0.82 | (0.77,0.86) | 786,342 | 964 | 0.91 | (0.85,0.97) | 539,271 | 713 | 0.98 | (0.91,1.06) |
| 38 | 10,809,805 | 8,130 | 3,057,049 | 1,664 | 0.72 | (0.68,0.76) | 1,791,154 | 1,059 | 0.79 | (0.73,0.83) | 1,198,083 | 781 | 0.87 | (0.80,0.93) |
| 39 | 19,697,143 | 9,642 | 5,578,125 | 1,833 | 0.67 | (0.63,0.70) | 3,176,033 | 1,110 | 0.71 | (0.67,0.75) | 2,138,471 | 800 | 0.76 | (0.71,0.82) |
| 40 | 13,932,683 | 6,156 | 3,444,795 | 1,295 | 0.85 | (0.80,0.90) | 1,904,999 | 775 | 0.92 | (0.85,0.99) | 1,277,952 | 542 | 0.96 | (0.87,1.04) |
| 41 | 4,192,974 | 2,182 | 1,031,087 | 420 | 0.78 | (0.70,0.86) | 573,647 | 253 | 0.85 | (0.74,0.96) | 368,822 | 181 | 0.94 | (0.81,1.09) |
| >=42 | 474,107 | 450 | 98,596 | 85 | 0.91 | (0.72,1.14) | 50,777 | 42 | 0.87 | (0.63,1.19) | 33,838 | 33 | 1.03 | (0.72,1.46) |
| Sweden | | | | | | | | | | | | | | |
| 37 | 84,054 | 94 | 19,140 | 23 | 1.07 | (0.68,1.69) | 15,435 | 14 | 0.81 | (0.46,1.42) | 3,705 | 9 | 2.17 | (1.09,4.30) |
| 38 | 214,584 | 118 | 64,535 | 29 | 0.82 | (0.54,1.22) | 52,110 | 21 | 0.73 | (0.46,1.16) | 12,425 | 8 | 1.17 | (0.57,2.39) |
| 39 | 356,537 | 151 | 113,069 | 37 | 0.77 | (0.53,1.10) | 92,000 | 26 | 0.67 | (0.44,1.01) | 21,069 | 11 | 1.23 | (0.66,2.27) |
| 40 | 410,385 | 148 | 149,712 | 46 | 0.85 | (0.61,1.18) | 119,808 | 37 | 0.86 | (0.59,1.22) | 29,904 | 9 | 0.83 | (0.42,1.63) |
| 41 | 255,560 | 103 | 109,031 | 40 | 0.91 | (0.63,1.31) | 84,377 | 31 | 0.91 | (0.61,1.36) | 24,654 | 9 | 0.91 | (0.45,1.78) |
| >=42 | 91,583 | 54 | 43,083 | 28 | 1.10 | (0.69,1.73) | 31,822 | 23 | 1.23 | (0.75,1.99) | 11,261 | 5 | 0.75 | (0.30,1.88) |

LGA babies compared to AGA babies born at the same gestational week

# **Additional references**

1.World Health Organization. ICD-10 : international statistical classification of diseases and related health problems : tenth revision 2004 [2nd ed:[Available from: <https://apps.who.int/iris/handle/10665/42980>.

2.World Health Organization. World Health Organization. International Classification of Diseases for Mortality and Morbidity Statistics (ICD-11 MMS) 2018 [ 11th ed:[Available from: [https://icd.who.int/browse11/l-m/en#](https://icd.who.int/browse11/l-m/en)!

3.Villar J, Giuliani F, Fenton TR, Ohuma EO, Ismail LC, Kennedy SH. INTERGROWTH-21st very preterm size at birth reference charts. Lancet. 2016;387(10021):844-5.

4.Villar J, Cheikh Ismail L, Victora CG, Ohuma EO, Bertino E, Altman DG, et al. International standards for newborn weight, length, and head circumference by gestational age and sex: the Newborn Cross-Sectional Study of the INTERGROWTH-21st Project. Lancet. 2014;384(9946):857-68.

5.Benchimol EI, Smeeth L, Guttmann A, Harron K, Moher D, Petersen I, et al. The REporting of studies Conducted using Observational Routinely-collected health Data (RECORD) statement. PLoS Med. 2015;12(10):e1001885.

6.The International Fetal and Newborn Growth Consortium for the 21st Century. INTERGROWTH-21st 2020 [Available from: <https://intergrowth21.tghn.org/>.
